# Clinical feasibility and preliminary outcomes of a novel mixed reality system to manage phantom pain: a pilot study

**DOI:** 10.1186/s40814-022-01187-w

**Published:** 2022-10-22

**Authors:** Thiru M Annaswamy, Kanchan Bahirat, Gargi Raval, Yu Yen Chung, Tri Pham, Balakrishnan Prabhakaran

**Affiliations:** 1 Department of Physical Medicine & Rehabilitation, Penn State Health Milton S. Hershey Medical Center, Hershey, PA USA; 2 Penn State Health Rehabilitation Hospital, Hummelstown, PA USA; 3 Simbe Robotics, South San Francisco, CA USA; 4 Physical Medicine & Rehabilitation Service, VA North Texas Health Care System, Dallas, USA; 5 Department of Computer Science, UT Dallas, Dallas, TX USA; 6 UT Southwestern Medical School, Dallas, TX USA

**Keywords:** Augmented reality, Phantom limb pain, Exercise therapy, Self-management

## Abstract

**Background:**

To assess the clinical feasibility of a virtual mirror therapy system in a pilot sample of patients with phantom pain.

**Methods:**

Our Mixed reality system for Managing Phantom Pain (Mr. MAPP) mirrors the preserved limb to visualize the amputated limb virtually and perform exercises. Seven patients with limb loss and phantom pain agreed to participate and received the system for 1-month home use. Outcome measures were collected at baseline and 1 month.

**Results:**

Four (of seven recruited) participants completed the study, which was temporarily suspended due to COVID-19 restrictions. At 1 month, in-game data showed a positive trend, but pain scores showed no clear trends. Functioning scores improved for 1 participant.

**Conclusions:**

Mr. MAPP is feasible and has the potential to improve pain and function in patients with phantom pain.

**Trial registration:**

Clinical Trials Registration, NCT04529083

**Supplementary Information:**

The online version contains supplementary material available at 10.1186/s40814-022-01187-w.

## Key messages regarding feasibility

- **What uncertainties existed regarding the feasibility?** The main uncertainties regarding the feasibility in this study of a home-based virtual mirror therapy program (Mr. MAPP) delivered to a sample of patients with lower limb amputation were (a) the ability to recruit patients meeting study criteria in our healthcare facility, (b) the ability to deliver the novel intervention at participants’ homes, (c) the appropriateness of assessment and outcome measures, and (d) the barriers faced by participants in receiving the intervention.
- **What are the key feasibility findings?** *Feasibility outcome data:*
  Recruitment ability: Of the 9 patients approached at the facility’s PM&R Amputee clinic, 7 consented to be enrolled in the study (recruitment rate of 78%). No participants met the exclusion criteria of motion sickness.
  Intervention deliverability: Four of seven participants fully completed the study (retention rate of 57%). Three patients withdrew from the study, with one citing lack of time, one finding no suitable location for Mr. MAPP, and one citing prosthesis pain. None withdrew due to therapy intolerance, adverse events, or dissatisfaction with Mr. MAPP.
  Outcome measure appropriateness: All 4 participants who completed the study reported satisfaction with the system and temporary relief of pain following therapy sessions with Mr. MAPP. One participant also reported benefit by using the system during episodes of phantom pain. No other participant self-initiated therapy sessions to relieve their pain.
  Barriers: No participant reported adverse events with exercises or the use of Mr. MAPP. Three participants required an additional home visit to assist with optimizing system setup, particularly with issues of camera positioning and Oculus sensor displacement. All participants were satisfied with the ease of use and expressed the desire to retain the system longer if possible.
- **What are the implications of the feasibility findings for the design of the main study?** This clinical feasibility pilot study demonstrated that exercises performed using virtual mirror therapy with the Mr. MAPP system are clinically feasible and Mr. MAPP shows potential in its ability to improve pain and physical functioning outcomes for patients with limb loss and phantom pain. Future fully powered, comparative trials between this system and standard-of-care approaches (including MT, pharmacological, and physical therapy interventions) are planned, which may help more definitively demonstrate the efficacy of this system in terms of pain and functional improvement. Additionally, we plan to evaluate the outcomes in self-directed treatment sessions as well as the potential benefits of longer trial durations. Finally, following these trials, we plan to develop a Mr. MAPP module for upper limb loss as well.

## Background

Phantom limb pain (PLP) is defined as a painful sensation perceived in an amputated part of the body [1, 2]. Chronic PLP remains one of the most traumatic consequences of amputation and affects 50–85% of amputees [3, 4]. Pain characterization, frequency, and stability of perceived PLP vary tremendously between patients, consequently creating challenges for effective treatment [5–8]. Unfortunately, there is a paucity of high-quality evidence supporting treatment efficacy (largely due to underpowered clinical trials) despite the plethora of potential therapeutic approaches to PLP [9–15]. A classic approach to addressing PLP is mirror therapy (MT) [16]. In MT, movement of an unaffected limb creates a reflective illusion of painless movement of the missing limb, thus mitigating PLP symptoms in some patients [16–20]. However, the static orientation of the mirror and the need for an unwavering focus on the illusion can create difficulties and result in suboptimal outcomes [21].

To overcome these barriers with traditional MT, various virtual and mixed reality-based solutions have been proposed [21, 22]. Virtual movements have been rendered onto a flat screen or a head-mounted display (HMD) to create the illusion of an intact limb through a variety of means. One method involves capturing affected limb movement and translating it into virtual limb motion in a virtual reality environment [23, 24]. Another involves superimposing intact limb motion into a projection of the missing limb using a top-down camera [25]. While other methods exist, similar to MT, many of these contemporary approaches also have challenges. For instance, they can reduce the sense of embodiment due to limb misalignment, artificial images or models, and discrepancies between real and virtual appearances [26, 27]. Furthermore, many systems have limited ability for environmental interactions and also require a plethora of body sensors which can hinder their use in non-supervised environments such as home settings. Most importantly, all currently existing systems are employed in a supervised healthcare environment—usually within a clinic or research lab setting. Several socioeconomic and logistical barriers may prevent routine clinical implementation of these systems. Users also may not be able to use them on demand for symptom management as many prefer [unpublished author observations], due to organizational/institutional limitations such as regular business hours. Due to these barriers, the feasibility and effectiveness of a self-guided, on-demand, mixed reality MT system have not been previously studied.

In order to address these obstacles in existing virtual systems, the authors have developed a *M*ixed *R*eality-based framework for *MA*naging *P*hantom *P*ain (Mr. MAPP) [28], which generates a real-time 3D model of the phantom limb by capturing and mirroring intact limb data with off-the-shelf cameras. Mr. MAPP uses an HMD, does not require body sensors, and allows phantom limb interaction with virtual objects, thereby providing an unencumbered experience with a high sense of embodiment within a home setting. Furthermore, unlike prior published work, in this study, the mixed reality MT system was also designed for home deployment and for use in an unsupervised, informal environment. These novel features to a previously existing framework provide the basis for a unique approach to PLP treatment, warranting a pilot study and feasibility evaluation. The knowledge accumulated from this trial and manuscript further informs the field of pain management and rehabilitation on PLP interventions and could guide future investigation into these methods and systems.

The primary goals of this clinical pilot study were to evaluate the feasibility and preliminary outcomes of a home-based virtual mirror therapy program delivered with the Mr. MAPP system in a sample of patients with lower limb amputation. We evaluated the following: (a) ability to recruit patients meeting study criteria in our healthcare facility, (b) ability to deliver the novel intervention at participants’ homes, (c) appropriateness of assessment and outcome measures, and (d) barriers faced by participants in receiving the intervention.

## Methods

### Mr. MAPP system overview

**Fig. 1:**
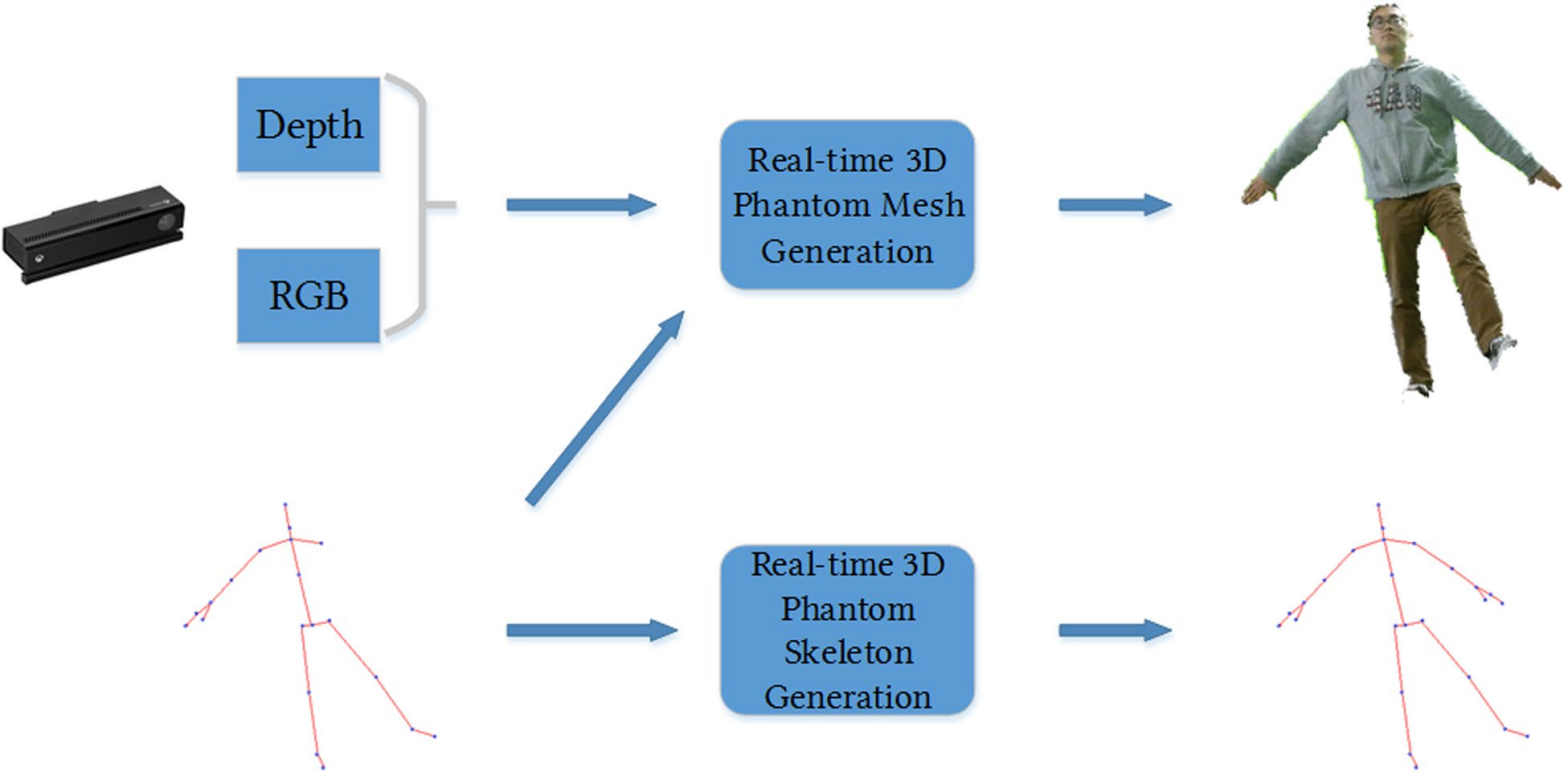
Pipeline of the Mr. MAPP framework [28]

The Mr. MAPP framework is designed to create a visual cue by generating a corresponding phantom limb in real time. It utilizes Microsoft’s Kinect V2 RGB-D cameras to capture the 3D avatar of the person, a laptop computer for running the games, and an Oculus Rift for visualizing the virtual environment (Fig. 1). The Kinect SDK uses a medial axis of symmetry to mirror the intact limb and Mr. MAPP uses the generated data to obtain a 3D point cloud, which allows for environmental interaction with the virtual limb. Mr. MAPP also utilizes a variety of post-processing adjustments to maintain an accurate lower limb illusion [29, 30]. A detailed summary of Mr. MAPP’s design and features has been previously published [29].

### Clinical pilot study

#### Setting, participants, and study criteria

The study was approved by our Institutional Review Board. Ten veterans who were established patients in the amputee clinic in our facility were targeted for recruitment in this study. Inclusion criteria include men and women, over the age of 18, with lower limb amputations (greater than 3 months post-surgery) who reported phantom limb pain of any duration or severity. Exclusion criteria include patients with open wounds or active infection in residual or contralateral limbs, history of seizures, visual or cognitive impairment that interferes with the ability to participate in a computerized exercise program, any active cardiac condition or an active medical issue that poses a contraindication to exercise therapy, lives more than 60 miles away from the Dallas Veterans Affairs (VA) Medical Center, and history of motion sickness induced by HMDs or immersive environment. Any patient experiencing motion sickness induced by HMDs during the therapy session was able to opt out of the study.

Established patients with lower limb amputations in our physical medicine and rehabilitation (PM&R) clinics were screened for inclusion in the study. Health Insurance Portability and Accountability Act (HIPAA) waivers were obtained for screening. Eligible participants were identified and underwent clinical evaluation by a physiatrist to confirm that they met the study criteria. After this evaluation, eligible patients were invited to participate, and those who provided written informed consent were enrolled into the study.

#### Study protocol and intervention

After consent was obtained, study participants were instructed on using the Mr. MAPP system in a single session. This consisted of completion of the baseline outcome instrument questionnaires, familiarization with Mr. MAPP, and formal instruction about the virtual MT exercise protocol. This one-time training session was conducted in the PM&R clinic and lasted approximately 1 h. Given that the key attributes of Mr. MAPP were its portability and ability to be used with minimal ongoing supervision, all participants were instructed in detail on how to use the system. Regardless of familiarity with technology prior to the session, all participants demonstrated an understanding of the Mr. MAPP system and were given an opportunity to ask any questions or clarify instructions. All the participants verbally confirmed that they understood how to use the software at the end of the session and were given the research team’s contact information for any later questions during the month.

The in-home virtual MT protocol was designed to emphasize patient convenience and minimize the need for supervised therapy. Regarding the implementation of these exercises in the software, Mr. MAPP was designed with the intention of registering large movements that were able to fit within the frame of the Kinect camera. Each exercise session consisted of three different exergames designed specifically for individuals with lower limb amputation and targeted three specific movements: (1) knee flexion and extension (Bubble Burst game) [28], (2) ankle dorsiflexion and plantarflexion (Pedal game), and (3) tandem bilateral lower extremity movement (Piano game) [29]. The exergames were standardized to keep the intervention consistent from participant to participant. The exercises/movements in the three exergames were designed by physical medicine and rehabilitation physicians and physical therapists’ input and were intended to target large lower extremity movements of hip flexion, knee extension, and ankle dorsi- and plantarflexion, which could be captured well by the camera and provided a satisfactory visual image of such movements in the amputated limb. Participants were instructed to play 2 exergame sessions daily for 1 month. Video demonstrations for reference were available as needed [31, 32].

The research team provided each participant with the Mr. MAPP system (including a laptop and camera) and assisted with home setup through an in-person visit (Supplemental material). The participant then used the system to perform their daily home exercises for 1 month to evaluate the sustainability of exercise behavior. At the end of this period, the system was returned. We decided on 1 month as the length of intervention for pragmatic reasons. We strove to attain a balance between giving each participant adequate time to become accustomed to the system and use it for its intended therapeutic purpose and taking into consideration the availability of a limited number of Mr. MAPP systems that could be simultaneously deployed for use by participants. Narrative ad hoc reports of participants’ experience with in-home and on-demand use were also collected to inform future clinical implementation of this system, a major goal of this pilot study. Throughout the course of the study, we kept an informal record of the difficulties that arose specifically as a result of the system needing to be used in the subject’s home.

#### Feasibility outcome measures

To assess recruitment ability, we calculated the percentage of all potentially eligible amputees (i.e., who reported suboptimal management of their PLP) that we were able to successfully recruit. To evaluate intervention deliverability, we calculated the retention among study participants. To assess outcome measure appropriateness, we assessed patients’ qualitative feedback on perceived intervention usefulness for their PLP, which would be compared to clinical measures. Finally, we documented the barriers and challenges faced by participants while using the in-home system. This data was collected through a user survey at the end of the 1-month intervention, which included questions regarding user interface experience with the software, overall satisfaction, review of ad hoc narrative reports, and an open-ended inquiry for any suggestions. Feedback from user surveys, other communications, and informal narrative reports were used to inform future modifications of this system including a specific focus towards in-home on-demand and sustainable use.

#### In-game data

Session time, duration, and gaming score were automatically recorded into a “digital diary.” Session time and duration served to assess the level of engagement and were used to verify the data in the participants’ self-reported exercise diary. The following attributes in the digital diary were analyzed: attendance patterns, performance improvement over time, and effect of session duration on the performance. Attendance patterns were also used to assess intervention feasibility by tracking adherence. In-game data also provided valuable insight into the effectiveness of an at-home system, primarily regarding the potential variability of real users in using the system as directed.

#### Clinical outcome measures

At the baseline and 1-month visits, study participants completed the McGill Pain Questionnaire (MPQ) and Patient-Specific Functional Scale (PSFS). The MPQ consists of three sections assessing pain characterization, change, and severity [33]. The PSFS quantifies activity limitation and measures functional outcome for patients with physical disabilities [34]. In this questionnaire, the participant is asked to identify up to three important activities they have difficulty performing due to their PLP and rate their level of difficulty. Change in their functional ability in these 3 self-identified activities was evaluated at each visit. Additionally, the participants were instructed to keep an exercise diary to track duration of their exercise sessions and to rate their average pain weekly on a Numerical Rating Scale (NRS) from 0 to 10.

Participants received weekly telephone support and online ad hoc technical support as needed. Adverse event data were also collected, and clinicians were informed for any clinical intervention as needed during this pilot study. Clinical study data were collected and managed using REDCap hosted at the local VAMC.

## Results

Ten participants were targeted in this clinical pilot study. However, due to restrictions imposed at the onset of the COVID-19 public health emergency (PHE), recruitment was temporarily halted at 7 participants. One participant already enrolled at the time of the PHE underwent her intervention as scheduled but completed her follow-up assessments virtually. Out of the 7 patients who consented to participate, 4 patients completed the entire study. Participants were mostly male (86%), the mean age was 64, and most were black (57%). Most had transtibial amputations (57%) that were nearly 1400 days old on average. Complete participant data is available in Table 1.

**Table 1:** Demographics, amputation history, and study participation status

Participant	Age range	Sex (male/female)	Race/ethnicity	Type of amputation	Time since amputation (days)	Study status
1	56–60	Male	White	Left below knee	1241	Failed to follow-up
2	50–55	Male	White	Left below knee	4678	Completed
3	70–75	Male	White	Right below knee	434	Participant declined
4	70–75	Male	Black	Right above knee	308	Completed
5	60–65	Male	Black	Left above knee	1297	Completed
6	60–65	Male	Black	Left below knee	1410	Completed
7	60–65	Female	Black	Left above knee	427	Pain due to prosthetic

### Feasibility outcome data

#### Recruitment ability

Of the 9 patients approached at the facility’s PM&R Amputee clinic, 7 consented to be enrolled in the study (recruitment rate of 78%). No participants met the exclusion criteria of motion sickness.

#### Intervention deliverability

Four of seven participants fully completed the study (retention rate of 57%). Three patients withdrew from the study, with one citing lack of time, one finding no suitable location for Mr. MAPP, and one citing prosthesis pain. None withdrew due to therapy intolerance, adverse events, or dissatisfaction with Mr. MAPP.

#### Outcome measure appropriateness

All 4 participants who completed the study reported satisfaction with the system and temporary relief of pain following therapy sessions with Mr. MAPP. One participant also reported benefit by using the system during episodes of phantom pain. No other participant self-initiated therapy sessions to relieve their pain.

#### Barriers

No participant reported adverse events with exercises or the use of Mr. MAPP. Three participants required an additional home visit to assist with optimizing system setup, particularly with issues of camera positioning and Oculus sensor displacement. Informal feedback revealed the need for scheduled check-ins from clinic personnel during early in-home use, including potentially virtual check-ins. All participants were satisfied with the ease of use and expressed the desire to retain the system longer if possible.

### In-game data

#### Attendance pattern

**Fig. 2:**
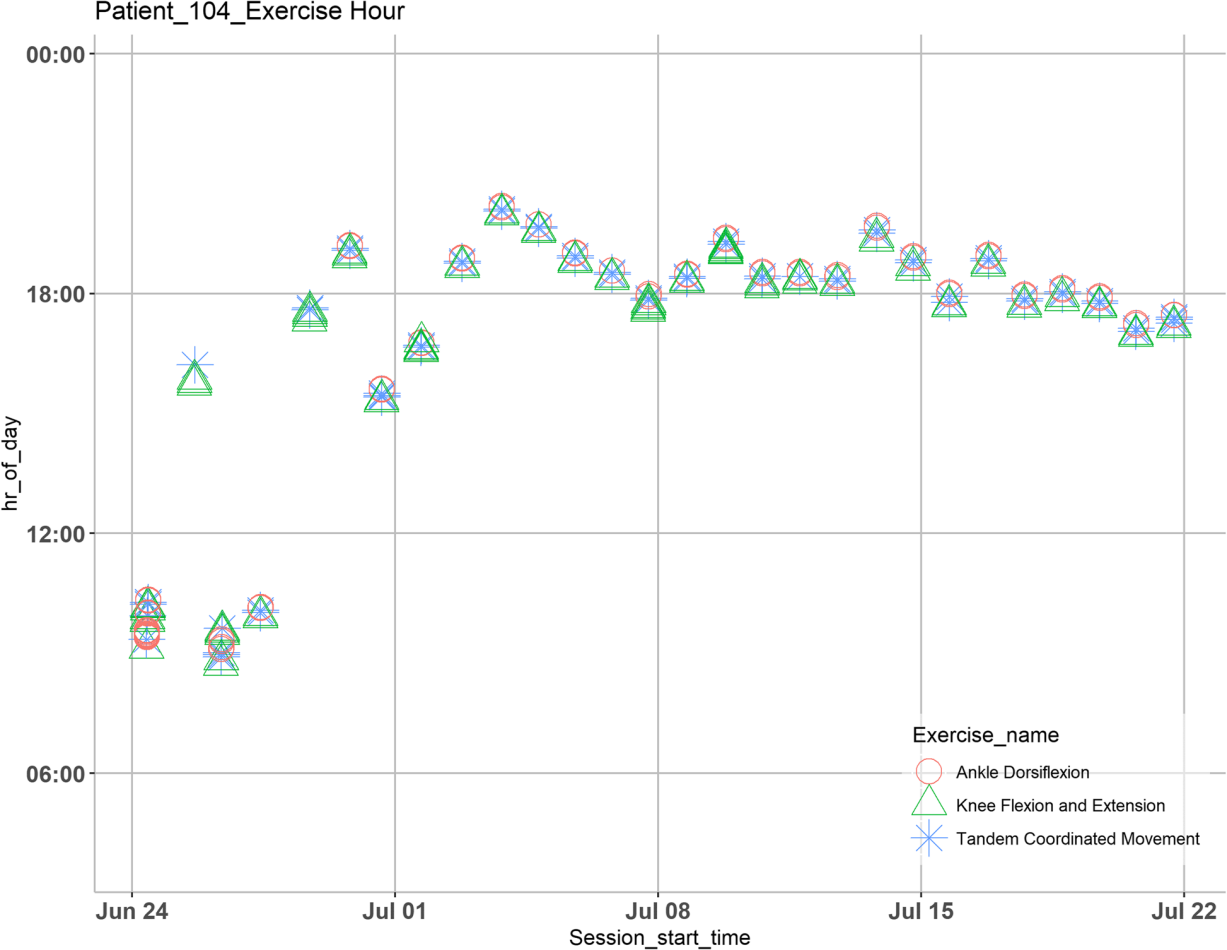
Analysis of time at which a certain game is played for a given day during the pilot study for participant #4

**Fig. 3:**
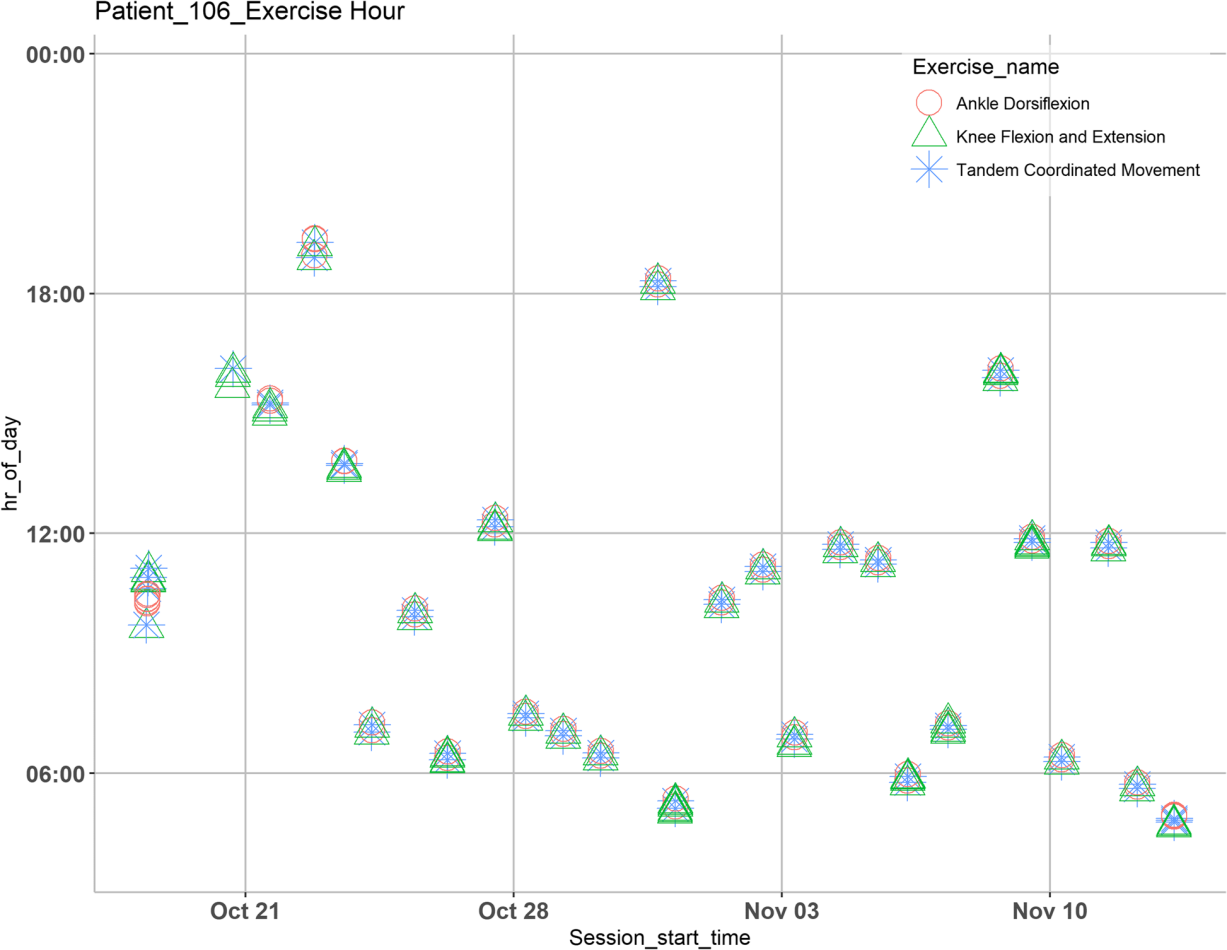
Analysis of time at which a certain game is played for a given day during the pilot study for participant #6

Half of our sample (2/4) had perfect adherence to the program. Participants #4 and #6 performed their exercise therapy sessions daily for 1 month, with #4 having a consistent start time while #6 having a varied schedule (Figs. 2 and 3). In contrast, digital diaries from participants #2 and #5 revealed sporadic participation in their sessions.

#### Performance

**Fig. 4:**
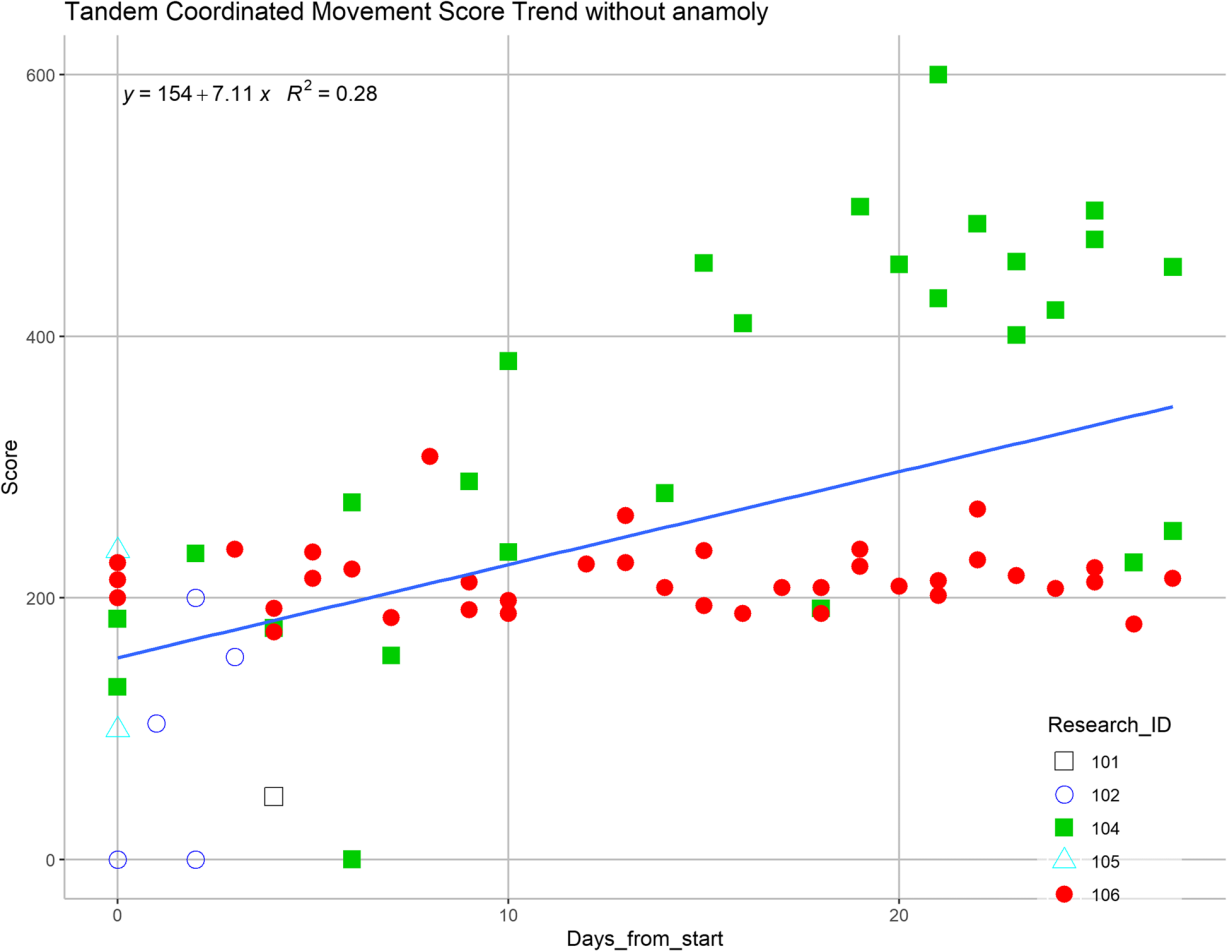
Analysis of scores over the study duration (participant #4 is labeled in solid green squares, and participant #6 is labeled in solid red circles) for tandem coordinated movement exergame

**Fig. 5:**
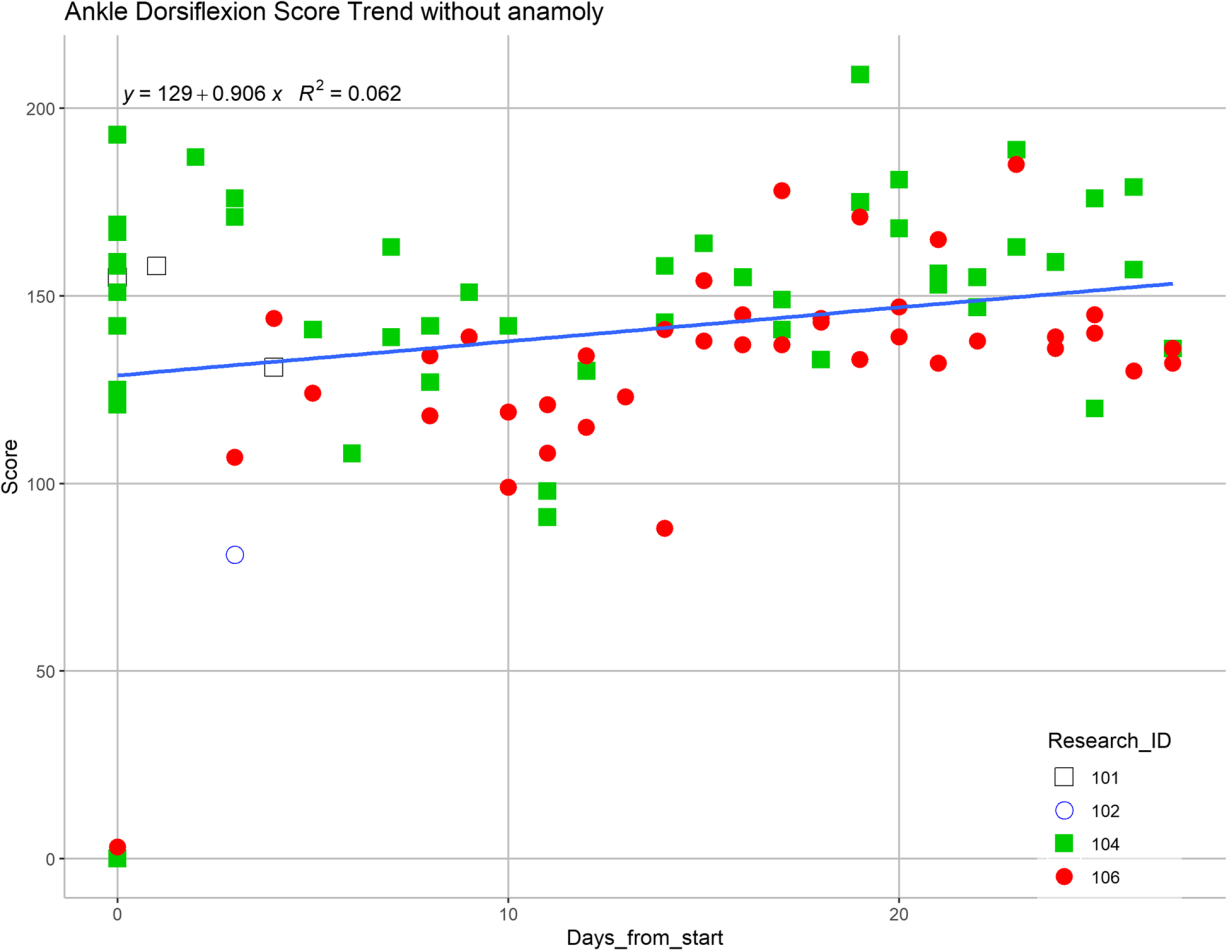
Analysis of scores over the study duration (participant #4 is labeled in solid green squares, and participant #6 is labeled in solid red circles) for ankle dorsiflexion exergame

Each participant’s game performance changes were variable, but a regression line based on all records for each game was drawn, illustrating a general trend towards improvement across all participants (Figs. 4 and 5). In the Piano game (Fig. 4), a positive correlation trend is observed in participant #4 but the trend was not observed in participant #6. In the Pedal game, a slight positive correlation was noted in all participants (Fig. 5). For the Bubble Burst game, the trend is neutral over time.

#### Session duration on performance

**Fig. 6:**
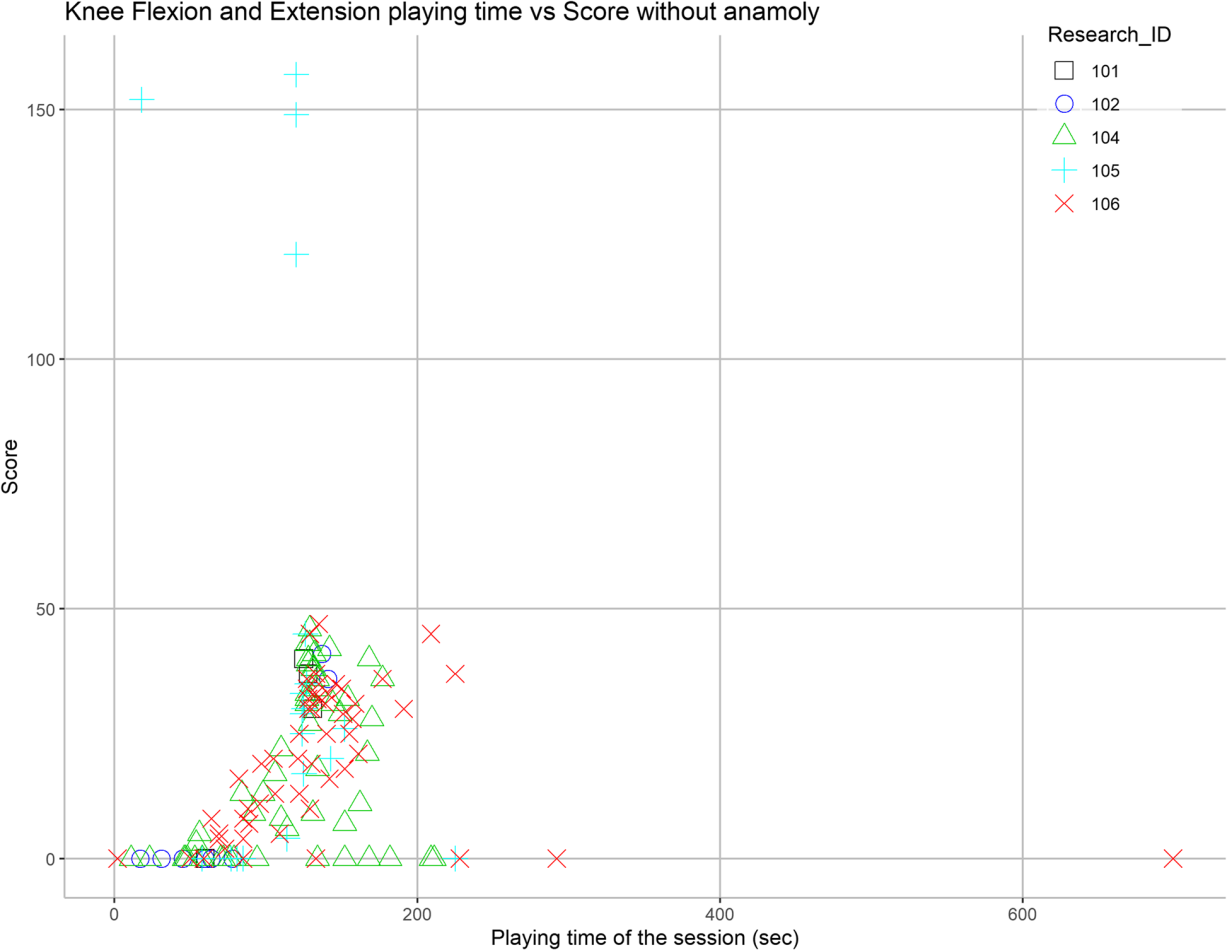
Effect of session duration on the performance for knee flexion and extension exergame

**Fig. 7:**
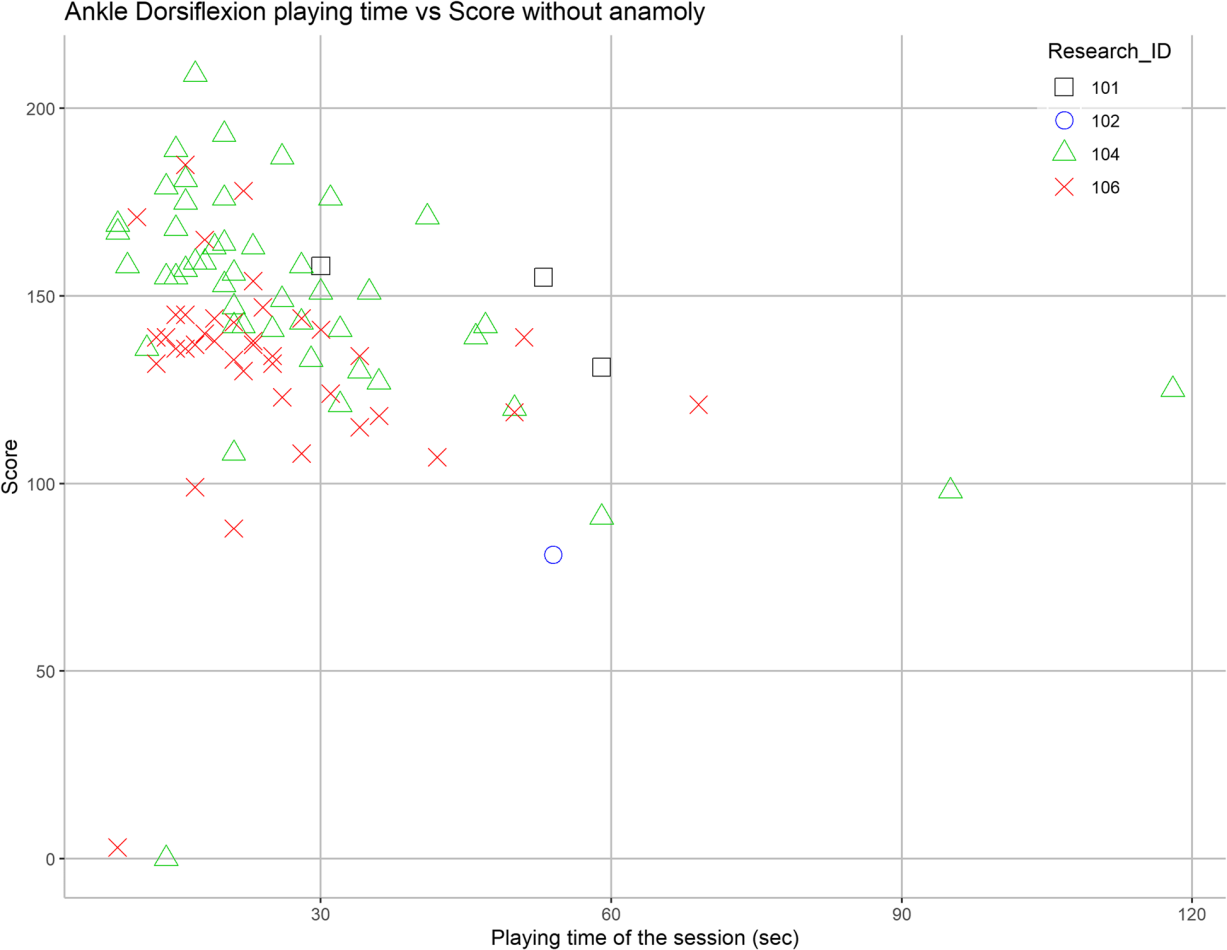
Effect of session duration on the performance for ankle dorsiflexion exergame

No clear correlation was noted in the relationship between performance and session duration (Figs. 6 and 7). The Bubble Burst game generally took 2–3 min to complete, while the Pedal game typically took under 40 s. The Piano game was not included in this analysis since it had a fixed duration of 2 min. Additionally, there were several instances of participants receiving a score of 0 despite significant play time and game launches.

### Clinical outcome data

Among the 4 participants who completed the 1-month clinical trial, no clear trend was noted in pain intensity as rated on the weekly NRS in the exercise diary (Table 2) or in pain changes in any of the 3 sections of the MPQ (Table 3).

**Table 2:** Exercise diary data

Exercise diary data				
Participant	Week	Average minutes exercised (per week)	Average minutes exercised (total)	Pain score (0–10)
2	Week 1	25.7	18.2	4
	Week 2	17.1		6
	Week 3	15.7		4
	Week 4	14.3		4
4	Week 1	21.6	15.4	7
	Week 2	12.7		7
	Week 3	13.7		6
	Week 4	13.7		
5	Week 1	47.1	42.9	0
	Week 2	38.6		1
	Week 3	40.7		1
	Week 4	45		
6	Week 1	40.5	28.5	3
	Week 2	27.9		2
	Week 3	23		2
	Week 4	22.7		1

**Table 3:** McGill Pain Questionnaire data

McGill Pain Questionnaire									
Participant	Visit	Section 1: What does your pain feel like? (0–78)	Section 2: How does your pain change with time?	Section 3: How strong is your pain?					
				Current pain strength (1–5)	Pain at its worst (1–5)	Pain at its least (1–5)	Pain of worst toothache (1–5)	Pain of worst headache (1–5)	Pain of worst stomach-ache (1–5)
2	Baseline	41	2	2	5	1	5	5	3
	1 month	40	2	2	3	1	3	4	4
4	Baseline	45	2	2	5	1		5	2
	1 month	67	2	1	5	2		5	2
5	Baseline	40	3	1	2	2	4	4	4
	1 month	38	3	1	2	1	4	4	2
6	Baseline	61	1	2	4	1	5	5	4
	1 month	58	2	1	5	1	5	4	4

Possible responses and key for “How does your pain change with time”:

1 = continuous and steady

2 = rhythmic period intermittent

3 = brief momentary transient

Participants exercised for an average duration ranging from 15.4 min per week (participant #4) to 42.9 min per week (participant #5), with pain scores that ranged at the low end between 0 and 1 for participant #5 and at the high end between 6 and 7 for participant #4.

PSFS data were available for only 3 participants (Table 4) because participant #2 was unable to identify functional activities they wanted to address. Each participant chose 2–3 activity-related goals. Participants #4 and #6 showed improvement in PSFS scores (#4: 6.33 to 7.67 and #6: 1 to 3.33), while participant #5 demonstrated a decrease in score (7.5 to 6.5). There was an apparent difference between overall baseline and 1-month PSFS scores (4.625 and 5.75, respectively).

**Table 4:** Patient-Specific Functional Scale data

Patient-Specific Functional Scale (PSFS)							
Participant	Baseline			1 month			Goals
	Goal 1	Goal 2	Goal 3	Goal 1	Goal 2	Goal 3	
4	7	6	6	8	7	8	1) Be able to stay standing while cooking 2) Ability to clean the house comfortably 3) Walking with crutches with more stability
5	6	9	-	7	6	-	1) Being able to play around with grandkids 2) Getting in and out of the shower
6	1	1	1	3	4	3	1) Walking is easier without PLP 2) Standing for long periods 3) Staying asleep without PLP disturbance

Range: 0–10

0 = unable to perform activity

10 = able to perform activity at the same level as before injury or problem

- = no goal set

Note: Participant #2 did not complete PSFS

## Discussion

The results of this study suggest that it is feasible to implement Mr. MAPP, a virtual MT intervention, to treat PLP in patients with lower limb amputation in a home environment. In addition, most reported satisfaction with the system, and a few reported temporary relief of symptoms with the use of Mr. MAPP. To the best of our knowledge, no other published reports on virtual MT systems used to treat PLP have implemented the system as a primarily in-home intervention [35–39].

Analysis of Mr. MAPP’s digital diary revealed that the improvements in performance primarily emerged from increases in repetition. This suggests that the gamified exercise therapy delivered with Mr. MAPP is challenging and can motivate the participants to improve their exercising behavior over time. While the sustainability of this behavior longer than the 1-month period evaluated in this study is unclear, the gamified experience may have the potential to increase patient engagement in self-management of in-home rehabilitative exercises.

The digital diary found varying levels of engagement in our sample, as demonstrated by inconsistent game activity by some participants. Technical factors and unique challenges faced as a result of in-home use of the system may have contributed to these findings. For instance, accommodating the camera position to a participant’s home may result in a suboptimal setup, leading to reduced quality of experience. Operating the gaming laptop may also pose challenges if participants did not have assistance. Because the device was typically 2 m away from the therapy area, participants would have to doff and re-don the HMD goggles to independently operate the laptop. Additionally, the digital diary detected occasional issues with 0-scored gaming sessions. Two possible reasons for this include (a) changes in Kinect, Oculus, or player orientation after initial home setup, which could cause misaligned or undeveloped virtual phantom limb and limit player interaction with the virtual environment, and (b) instability of the teleportation procedure responsible for centering the player before each game. While participants can typically self-adjust to correct the issue, this discrepancy cannot be corrected if the misplacement is in the vertical axis. Additionally, the 0-score data anomalies may have contributed to the lack of correlation between game performance and session duration. Optimizing all of these technical and environmental considerations is paramount to maximize patient engagement and quality of experience for participants, especially for fully powered in-home clinical trials with Mr. MAPP in the future.

Additionally, review of digital diary records suggest that participants who completed the study were able to adhere to their recommended prescription. While occasional discrepancies were noted between digital and paper diaries, it likely may be due to recall error. Therefore, integration of manual diary entries into the digital interface may minimize potential inconsistencies.

Although our study participants did not show clear trends in pain improvement assessed on the MPQ and the NRS, this feasibility study was not powered to determine efficacy. Nevertheless, all 4 participants reported temporary improvements in pain after therapy sessions. Additionally, one participant reported benefit in using the system to self-initiate treatment during episodes of PLP. To our knowledge, no prior virtual MT therapy studies have assessed the utility of ad hoc self-initiated treatment sessions in the long-term management of PLP, possibly due to its episodic nature. Given this instance of successful self-directed treatment, further research is necessary to explore the potential utility and accessibility of this therapy modality, as it may serve as a safe and feasible option for patients.

Analysis of PSFS scores indicated significant functional gains over 1 month with the use of the Mr. MAPP system, suggesting that by alleviating pain-related interference with functioning there is an opportunity to improve in physical functioning in patients with PLP. As we design the future clinical trial with Mr. MAPP, we plan to study pain interference with function by using the Brief Pain Inventory (BPI) scale, as recommended by a recent VA Chronic Musculoskeletal Pain Research Work Group [40].

Evaluation of innovative, non-pharmacological treatment options for chronic pain conditions is one of the VA’s core priorities as outlined in a recent State of the Art Conference [41]. Improving the quality of life and function in this patient population without the additional requirement of trained healthcare personnel and with a minimal additional cost of care can provide significant value. Furthermore, Mr. MAPP-facilitated home exercise therapy can potentially mitigate the increased utilization of rehabilitation services that is often observed in this population.

Future fully powered, comparative trials between this system and standard-of-care approaches (including MT, pharmacological and physical therapy interventions) are planned, which may help more definitively demonstrate the efficacy of this system in terms of pain and functional improvement. These trials should include a comparison group of individuals who are participating in traditional physical therapy with MT to better assess head-to-head effectiveness with the current standard of care for those with PLP. Additionally, we plan to evaluate the outcomes in self-directed treatment sessions as well as the potential benefits of longer trial durations. Studying better transitions and hand-offs between clinic/hospital-based treatment to in-home management may also help inform the practical implementation of such interventions. The next steps would also include a thorough evaluation into both the direct and indirect costs of the Mr. MAPP system and its subsequent commercial applications. Finally, following these trials, we plan to develop a Mr. MAPP module for upper limb loss as well.

### Limitations

This was a clinical feasibility and pilot evaluation of a small sample of patients with PLP, meaning this study was not powered to measure clinical efficacy. While some minor trends in clinical improvement in pain and functional outcomes were observed, caution should be exercised to avoid over-interpreting these outcomes. Due to the lack of a control group, we cannot definitively attribute the cause of the observed outcomes to the system. Several factors that may influence outcomes were not controlled for in this study, including duration and characteristic of PLP, etiology and level of amputation, and minutes of exercise. Although participants did show increased engagement over time and increasing motivation to perform prescribed therapy was an objective of the intervention, motivation was not directly evaluated in this study. Additionally, pre-defined outcomes were not used to determine if feasibility criteria were met, which will be addressed in future studies. Despite these limitations, this clinical feasibility pilot study did demonstrate that exercises performed using virtual MT with the Mr. MAPP system are clinically feasible and show potential in its ability to improve pain and physical functioning outcomes for patients with limb loss and PLP.

## Conclusions

Using Mr. MAPP, a novel and gamified virtual mirror therapy system to perform in-home exercises by community-dwelling patients with limb loss and phantom limb pain, is feasible and has shown the potential to improve pain and pain-related functional outcomes in patients. Further research including a fully powered prospective study with appropriate control is needed to better evaluate its efficacy in improving outcomes compared to traditional approaches.

## Supplementary Information

**Additional file 1.** In-home Setup.

## Data Availability

The datasets used and/or analyzed in the current study are available from the corresponding author on reasonable request.

## Abbreviations

PLP: Phantom limb pain
MT: Mirror therapy
HMD: Head-mounted display
Mr. MAPP: Mixed reality system for Managing Phantom Pain
3D: 3 dimension
RGB-D: Red-green-blue-depth
SDK: Software development kit
VA: Veterans Affairs
PM&R: Physical medicine and rehabilitation
HIPAA: Health Insurance Portability and Accountability Act
MPQ: McGill Pain Questionnaire
PSFS: Patient-Specific Functional Scale
NRS: Numerical Rating Scale
REDCap: Research electronic data capture
COVID-19: Coronavirus disease-19
PHE: Public health emergency
BPI: Brief Pain Inventory
