# Supplementary material for: Clinical feasibility and preliminary outcomes of a novel mixed reality system to manage phantom pain: a pilot study
Source: Pilot Feasibility Stud. 2022 Oct 22;8:232. doi: 10.1186/s40814-022-01187-w (PMC9588245; doi:10.1186/s40814-022-01187-w)
Supplement: Supplementary file 1 — Additional file 1. In-home Setup. [file 40814_2022_1187_MOESM1_ESM.docx]

*In-Home Setup:* The following process was followed for the one-time in-home set up for study participants. First the research team member ensured that there was enough free space available for the participant to move (approximately 3 x 5 meters) during the exercises. A safe boundary area using the ‘Guardian System’ provided by Oculus was enabled during this set-up visit. Second, a flat table surface (approximately 1.5 x 0.5 meters) was needed for securing a pair of Oculus Rift sensors, a Kinect camera and a 17" laptop. When a suitable surface was not available, a foldable table was provided, and sensors were secured separately around the laptop. The Kinect camera was placed around 1-meter height from the ground and the participant was instructed to sit on a chair approximately 2.5-3 meters in front of the camera. Third, the participant was instructed to wear the HMD and experience the virtual environment and the game. Additional adjustments were then made of the position parameters of the avatar in the virtual environment if the camera could not be secured in the ideal position. Fourth, participants were instructed on two separate interfaces: a) a webpage on laptop for launching each game and b) the game scene on the HMD. Given that the two interfaces were on separate devices, the research team member would instruct another in-home member (if any) as an assistant to help operate the laptop when the participant is wearing the HMD. If no assistant was available, the participant was instructed on handling both interfaces themselves. In the game scene, the participant would use the button on Oculus controller to start playing; the system would then calibrate the user's position by teleporting their avatar to the center of the gaming area in the virtual environment. Also, the participant could refine their positioning in the virtual environment after teleportation by moving their chair slightly if needed. However, they were advised to not make any larger movements for safety reasons. Finally, each game was launched and run-through once during this set-up process to ensure that everything was in working order. Typically, this process took about 1 to 2 hours, which included around 25 mins for device setup and rest of the time to review all the details and answer any questions about the game and the system. Also, a printed handout was provided as a reference
